# Acceptance Factors of Mobile Apps for Diabetes by Patients Aged 50 or Older: A Qualitative Study

**DOI:** 10.2196/med20.3912

**Published:** 2015-03-02

**Authors:** Madlen Scheibe, Julius Reichelt, Maike Bellmann, Wilhelm Kirch

**Affiliations:** ^1^Technische Universität DresdenMedizinische Fakultät Carl Gustav CarusResearch Association Public Health Saxony and Saxony-AnhaltDresdenGermany

**Keywords:** mobile apps, mobile health, elderly, diabetes mellitus, blood sugar self-monitoring, patient acceptance of health care, qualitative research, guided interviews

## Abstract

**Background:**

Mobile apps for people with diabetes offer great potential to support therapy management, increase therapy adherence, and reduce the probability of the occurrence of accompanying and secondary diseases. However, they are rarely used by elderly patients due to a lack of acceptance.

**Objective:**

We investigated the question “Which factors influence the acceptance of diabetes apps among patients aged 50 or older?” Particular emphasis was placed on the current use of mobile devices/apps, acceptance-promoting/-inhibiting factors, features of a helpful diabetes app, and contact persons for technical questions. This qualitative study was the third of three substudies investigating factors influencing acceptance of diabetes apps among patients aged 50 or older.

**Methods:**

Guided interviews were chosen in order to get a comprehensive insight into the subjective perspective of elderly diabetes patients. At the end of each interview, the patients tested two existing diabetes apps to reveal obstacles in (first) use.

**Results:**

Altogether, 32 patients with diabetes were interviewed. The mean age was 68.8 years (SD 8.2). Of 32 participants, 15 (47%) knew apps, however only 2 (6%) had already used a diabetes app within their therapy. The reasons reported for being against the use of apps were a lack of additional benefits (4/8, 50%) compared to current therapy management, a lack of interoperability with other devices/apps (1/8, 12%), and no joy of use (1/8, 12%). The app test revealed the following main difficulties in use: nonintuitive understanding of the functionality of the apps (26/29, 90%), nonintuitive understanding of the menu navigation/labeling (19/29, 66%), font sizes and representations that were too small (14/29, 48%), and difficulties in recognizing and pressing touch-sensitive areas (14/29, 48%). Furthermore, the patients felt the apps lacked individually important functions (11/29, 38%), or felt the functions that were offered were unnecessary for their own therapy needs (10/29, 34%). The most important contents of a helpful diabetes app were reported as the ability to add remarks to measured values (9/28, 32%), the definition of thresholds for blood glucose values and highlighting deviating values (7/28, 25%), and a reminder feature for measurement/medication (7/28, 25%). The most important contact persons for technical questions were family members (19/31, 61%).

**Conclusions:**

A lack of additional benefits and ease of use emerged as the key factors for the acceptance of diabetes apps among patients aged 50 or older. Furthermore, it has been shown that the needs of the investigated target group are highly heterogeneous due to varying previous knowledge, age, type of diabetes, and therapy. Therefore, a helpful diabetes app should be individually adaptable. Personal contact persons, especially during the initial phase of use, are of utmost importance to reduce the fear of data loss or erroneous data input, and to raise acceptance among this target group.

## Introduction

Numerous mobile apps exist that aim to support the self-management of patients with type 1 and type 2 diabetes. In addition to the documentation of blood glucose values, such apps are able to graphically depict those values, offer an analysis of trends, provide additional information about the disease, or to share relevant data with the attending physician [1]. Hence, they offer great potential to support therapy management, increase therapy adherence, and reduce the probability of accompanying and secondary disease occurrence.

Currently, 387 million people aged 20 to 79 years suffer from diabetes worldwide. This number is expected to rise to 205 million people by the year 2035. The prevalence of diabetes varies between the continents, with 5.1% in Africa and 11.4% in North America and the Caribbean [2]. The amount of undiagnosed cases is estimated to be between 27.1% and 53.6%. In 2014, 4.9 million people died from diabetes-related complications. In Germany, the country where this study was conducted, diabetes prevalence is currently at 9.0%, and more than 95.0% of those are suffering from type 2 diabetes [3]. This type of diabetes typically occurs at an advanced age and is the result of an interaction between genetic predisposition and environmental factors, especially physical inactivity, malnutrition, and the resulting obesity from these factors. The result is a decreased level of insulin action (ie, insulin resistance) and release. Contrary to type 2 diabetes, type 1 diabetes is an autoimmune disorder leading to decreased insulin release until there is a complete lack of insulin as a result of the destruction of the insulin-producing cells [4].

People aged 50 or older suffer disproportionately from diabetes mellitus, particularly type 2 diabetes [4]. However, as shown in the recently released Diabetes App Market Report, very few patients of this target group utilize diabetes apps to support their treatment [5]. Previous studies confirmed that the below-average utilization of such apps is caused by a lack of acceptance within the target group [5-10]. Therefore, we investigated the following question within the scope of our study: “Which factors influence the acceptance of diabetes apps among patients aged 50 or older?” We placed particular emphasis on the current usage of mobile devices and apps, acceptance-promoting/-inhibiting factors, features of a helpful diabetes app, and contact persons for technical questions. This study was the third of three substudies we conducted investigating the factors that influence acceptance among diabetes patients aged 50 or older. The two other accompanying substudies were previously published by Arnhold et al in April 2014 [1].

Several studies have focused on factors influencing the acceptance of technology, the most prominent being the Technology Acceptance Model (TAM) by Davis [11]. This model was used as a foundation for subsequent acceptance models and studies, due to its proven explanatory power. These subsequent models focused on individual target groups, technologies, or specific cases of application [6-10,12-16]. From there, new models and theories emerged, such as the Mobile Phone Technology Acceptance Model (MOPTAM) by Kwon and Chidambaram [17], the Unified Theory of Acceptance and Use of Technology (UTAUT) by Venkatesh et al [18], and the Senior Technology Acceptance and Adoption Model (STAM) by Renaud and van Biljon [19].

However, none of the previously developed models or studies examined the factors influencing the acceptance of mobile apps for diabetes among patients aged 50 or older. And in terms of the superordinate topic of acceptance of mobile health apps among patients aged 50 or older, only one related study was found at the time of preparing this article [16].

Therefore, we first had to test the existing models and their influencing factors against their applicability and relevance to the research questions investigated here. Additionally, we consulted studies, guidelines, and standards—International Organization for Standardization (ISO) and Deutsches Institut für Normung e.V. (DIN)—in the planning phase, taking into account their research foci, as shown in Multimedia Appendix 1 [20-47]. These studies helped provide the theoretical background for this study.

## Methods

### Overview

To investigate relevant acceptance factors, we chose the qualitative method of guided interviews, which added a qualitative dimension to the two prior quantitative substudies. Our aim was to understand the subjective perspective of older diabetes patients toward apps. We consciously opted for a personal approach with the study participants using personal interviews instead of questionnaires. This approach was used since a certain degree of insecurity toward the research topic was to be expected among the participants, due to a possible lack of previous experience. This approach enabled us to adapt the interview guideline according to the individual survey participants and their previous experiences in handling smartphones, tablets, and apps. Furthermore, the assessment of factors promoting or inhibiting the acceptance of diabetes apps by the elderly is a complex issue, and a qualitative approach was better suited to examine this topic. This study was approved by the Ethics Committee of the Technische Universität (TU) Dresden (reference number: EK 241072013), prior to the initiation of interviews. The interviews were conducted between July and December of 2013.

### Interview Guideline

We developed a theory-based and uniform interview guideline with open-ended questions as a basis for the study (see Multimedia Appendix 2). It provided the foundation for the comparability of the answers. At the end of each interview, we asked the patients to test and evaluate two existing diabetes apps to reveal difficulties in (first) use:

1. *OnTrack Diabetes* version 2.8.8 (Medivo) for the Android 4.4.2 mobile operating system tested on a Samsung Galaxy Note 10.1.
2. *Glukose Monitor* version 2.7 (Taconic System LLC) for the iOS 7.0 mobile operating system tested on an iPad (4^th^ generation).

**Figure 1:**
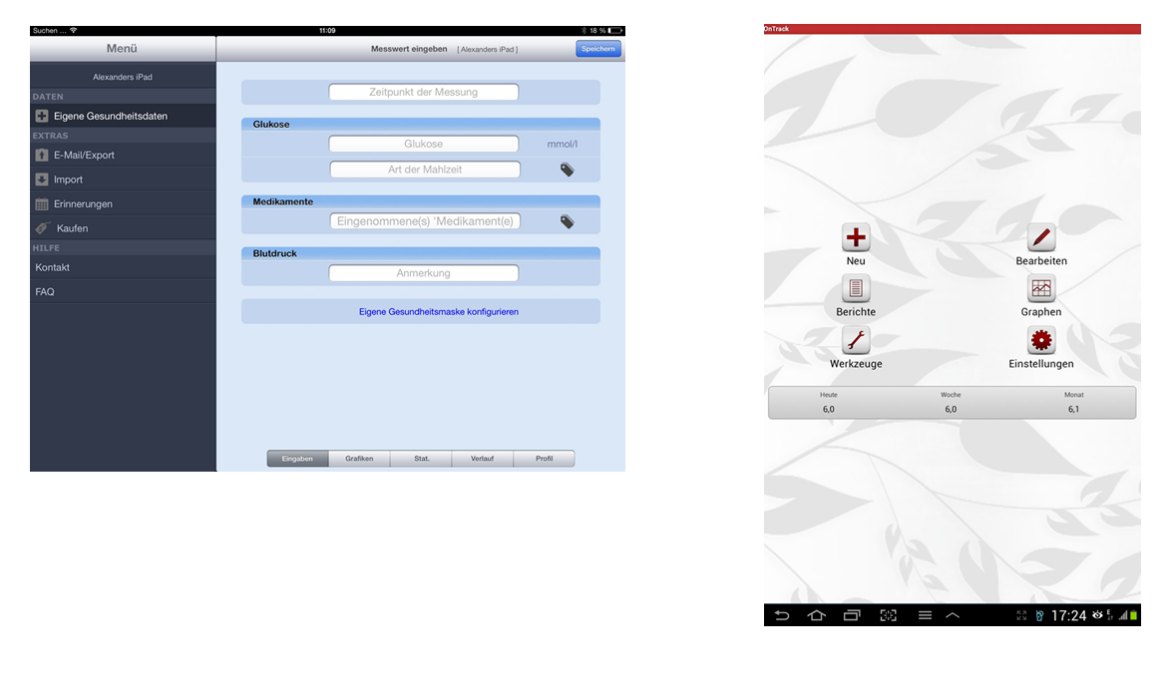
Screenshots of the start screens of the tested apps, Glukose Monitor (left) and OnTrack Diabetes (right).

The tested apps needed to satisfy the following criteria: (1) have German content, (2) be among the top 10 most commonly installed diabetes apps in the respective app store at the time of their selection (July 2013), and (3) be multifunctional (ie, able to combine several functions within one app). The following functions were offered by both apps: documentation function, analysis function, reminder function, and data forwarding/communication function. These functions are described in more detail in Arnhold et al [1]. Figure 1 shows screenshots of the start screens of both apps in order to illustrate their range of functions. In the run-up to the test, the participants did consciously not receive any form of introduction to the apps or the devices on which they were presented. The order in which the apps were presented was randomized to prevent an impact by the presentation order. Both apps were tested on tablets to increase the user-friendliness for the target group [48]. A pretest of the interview guidelines was performed prior to the commencement of the field work. Based on the results of the pretest, the guideline was slightly revised.

### Recruitment of Subjects

We decided to include both type 1 and type 2 diabetics in the study, as currently available apps address both types equally and rather differ in terms of the range of functions. Additionally, we included both participants with and without prior experience with mobile devices and apps to include the perspectives of both groups. The test subjects were recruited from diabetics’ self-help groups, diabetics’ associations, specialty shops for diabetics, general medical practices, diabetologists’ practices, and pharmacies. The following inclusion criteria were defined: (1) aged 50 years or older, (2) diagnosed with type 1 or type 2 diabetes mellitus (via patients’ self-disclosure), and (3) sufficient cognitive abilities to participate in a 60-minute interview. Persons who did not meet the inclusion criteria were excluded from this study.

### Data Evaluation

All interviews were transcribed verbatim. The transcripts were the foundation upon which consecutive data analysis was performed. To analyze the data, we used the structured content analysis by Mayring [49], which allows for an association between the deductive and inductive creation of categories [49,50]. The analytical focus was on designing a system of categories and subcategories, as well as their characteristics [49], which in turn served as a structural dimension. We started the analysis in accordance with the structure of the guideline [50]. The interview questions were partially transcribed directly into analysis categories. Based on the content, all relevant text passages were extracted and put into their specific categories [50]. Developing the categorical system and the deductive category application represented the initial framework with which we structured the content and the analysis. During the process of examining and analyzing the individual interviews, we tested, modified, and specified the categorical system. Missing, but relevant, categories were developed and added inductively based on the text material that we collected. This was done up to the point at which the system became saturated (ie, no new categories emerged) [50]. This method complies with the principle of openness within qualitative research.

The chosen data collection method enabled us to quantitatively analyze the data due to its systematic and rule-based approach. The individual steps were processed with the qualitative data analysis (QDA) software MAXQDA (2011 version).

The following steps briefly explain the specifics of the method used for analyzing the gathered data:

1. We assigned individual categories to interview sections if the category was clearly relevant for a certain part of the interview, or if the relevance became clear in conjunction with other interview sections.
2. Within a single interview, each category was allocated only once. If a certain aspect was presented multiple times by a participant, it was considered as being mentioned only once to prevent the evaluation from being unbalanced.
3. Overall, we interviewed 32 people with diabetes. The method we chose allowed us to individually adapt the interview guideline to the actual interview, and to the aspects presented as most relevant by the participants. In turn, this resulted in interviews where we did not ask all possible questions, had some unanswered questions, or where unexpected statements were added by the participants themselves. Therefore, the previously mentioned sample of 32 participants is not necessarily equal to the number of interviews and answered questions used for data analysis. The sample number is equal, however, to the number of participants who addressed the individual topics.

## Results

### Overview

Overall, we interviewed 32 people with diabetes aged 50 or older. The recommended sample size for guided interviews is set at 30 people [51]. The interviews lasted between 15 and 90 minutes. The following represents a selection of the survey results. An overview of all results can be found in Multimedia Appendix 3.

### Sociodemographics

There were an equal number of female (16/32, 50%) and male (16/32, 50%) participants in the interviews. The mean age of participants was 68.8 years (SD 8.2). Of all the participants, 44% (14/32) successfully completed vocational education, 13% (4/32) held a degree from a technical college, and 34% (11/32) had a university degree. Within their last or current job positions, 81% (26/32) had been working as employees, 13% (4/32) had been self-employed, and 6% (2/32) were employed as skilled workers. The majority of participants (17/32, 53%) were between 65 and 74 years of age. Of all the participants, 78% (25/32) have had diabetes mellitus for more than 10 years. Of the participants, 66% (21/32) had type 2 diabetes, 31% (10/32) had type 1 diabetes, and 1 patient out of 32 (3%) was afflicted with a hybrid form of both type 1 and type 2 diabetes.

### Interest in New Technologies for Diabetes Treatment and Current Usage

Of all the participants, 34% (11/32) described themselves as highly interested in new technologies. Of the participants, 53% (17/32) were open-minded in terms of technology, provided that it entails an additional benefit to their treatment, such as having a positive effect on their therapy/blood glucose values or improved convenience of self-management. Only 13% (4/32) of the interviewed diabetics described themselves as having no interest in innovative technologies, whatsoever.

At the time of the survey, 25% (8/32) of the interviewed diabetics older than 50 years of age owned a smartphone or tablet and 47% (15/32) knew apps. However, only 2 diabetics out of 32 (6%) already used a diabetes app for the purpose of documentation of blood glucose values, namely the *OnTrack Diabetes* app for Android (Medivo) and the *DiabetesPlus für Typ 2-Diabetiker* app for Android and iOS (SquareMed Software GmbH).

### Reasons Against Using Smartphones, Tablets, and Apps

Within this study, the essential aspects influencing the acceptance of diabetes apps were the self-reported reasons for or against the utilization of mobile devices and apps, as well as the obstacles that emerged during the actual app test. The following sections will describe the most influential factors in more detail.

We decided to consider the reasons against using a smartphone, tablet, and apps in one section, as the use of a portable device is the essential prerequisite to assessing apps. Additionally, the principles in handling these devices are similar. The main obstacles during the app test were a lack of additional benefits—for smartphones/tablets (10/18, 56%) and apps (4/8, 50%)—as well as finding the initial training and the handling phase for smartphones and tablets to be too complicated (6/18, 33%). One participant quoted the following:

As long as the alternative doesn´t provide me with a technical advantage or true advantage, I won´t put any efforts into mastering this, I mean, a smartphone requires a certain amount of practice, so yeah, I haven´t gotten around to doing this as I don´t see the personal advantage.

Participant 2

Out of 18 participants, 4 (22%) of them stated that the financial cost-to-benefit ratio of smartphones/tablets was unacceptable, especially when the device would only be used for diabetes treatment. Of 18 participants, 5 (28%) had concerns regarding the protection of their private data, as illustrated in the following quote:

...an inhibitional threshold where one could make a mistake and that data, personal data, could get lost, or that any involuntary payments might be necessary that were hidden somehow.

Participant 1

Additionally, the interviewed diabetics were concerned with a lack of interoperability between different devices and apps both for smartphones/tablets (2/18, 11%) and apps (1/8, 13%).

### Issues Encountered During App Tests

**Figure 2:**
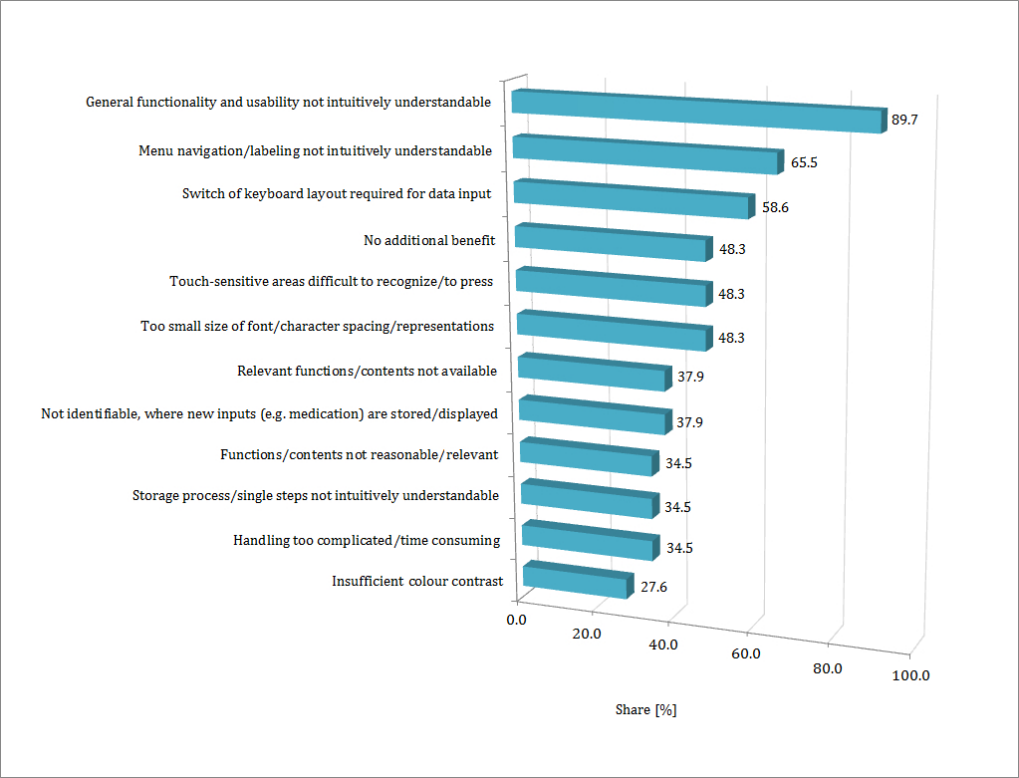
Issues encountered during app tests (multiple selections possible, n=29).

In addition to the self-reported obstacles, an app test was conducted to determine obstacles during the actual practical use of mobile devices and apps, which in some cases was the very first contact with such technology. On average, the participants tested each of the two apps for 11 minutes (22 minutes in total). Participants without previous experience spent more time on testing (26 minutes on average) compared to those who already used a smartphone or tablet (19 minutes on average). Figure 2 presents the most common obstacles. They were either observed by the interviewer or self-reported by the interviewees. Of the 32 participants, 3 (9%) were not able to participate in the app test section, as the interviews were conducted via telephone.

Out of the remaining 29 participants, 26 (90%) felt that neither functionality nor usability of the apps were intuitive or easy to grasp. This was especially true for those with no prior experience, who required additional aid (eg, how to enter data into the app). The following is an excerpt from transcript of the interview between interviewer and participant:

Would you spontaneously know what to do here? Hmm?

Interviewer

No, not spontaneously.

Participant 5

No?

Interviewer

Nope.

Participant 5

Could you enter data here by yourself?

Interviewer

No, but I should know how to do it.

Participant 5

Help was requested as to how to make the keyboard appear only after touching sections that ask for data to be entered, which wasn´t the case with previous generations of mobile devices. This is illustrated in the following interview excerpt:

And now, where to put this data?

Participant 12

It would go here. You are now supposed to touch here and then you can enter it.

Interviewer

Then the keyboard appears, hmm.

Participant 12

An additional difficulty was in understanding the symbols and functions of the keyboard, which were not intuitive and complicated the process of entering data even more. For the sake of completeness, it is important to mention that the difficulties encountered while using an onscreen keyboard cannot be counted as app-specific issues, as this may be the result of a lack of experience with smartphones and tablets, in general.

Of the 29 participants, 19 (66%) felt insecure and uncertain in terms of navigating through the menu within the apps, especially when switching between different layers of the menu. This is illustrated in the following interview excerpt:

If I want to go back, I´d simply have to click =.

Participant 23

Abort.

Interviewer

= ah on abort. Aha. And then again, again back or =.

Participant 23

Exactly. You can swype. If you want to go up.

Interviewer

= alright. So that´s how this works. This is a little different compared to my own.

Participant 23

Exactly.

Interviewer

There are keys or something, where I can go back or this arrow. Alright. This works by swype back and forth right?

Participant 23

Correct.

Interviewer

The participants faced issues such as adapting to different devices, operating systems (Android vs iOS), or apps and their individual layouts. This was particularly the case when attempting to save the progress on different devices, and this led to insecurity for 34% (10/29) of participants. Occasionally, it resulted in data loss. This issue is illustrated in the following interview excerpt:

Now that I saved it, I must be able to find my data again. History? Now, I have also entered my medication. Where do I find this?

Participant 1

Well, you could try this up here, but ahm...

Interviewer

Well, they´re nowhere to be found. Did I forget to save them, I thought I had saved them?

Participant 1

Of 29 participants, 14 (48%) had difficulty in identifying and pressing touch-sensitive areas on the touchscreen. Another obstacle presented itself when users had to switch keyboard layouts, between numbers and letters, to enter data into the app. This also led to insecurity for 59% (17/29) of the participants. Criticism was also voiced about the small size of font, the space between letters and representations (14/29, 48%), the insufficient color contrast (8/29, 28%), and the inability to flexibly adapt the size of the font and representations to individual needs (4/29, 14%). Moreover, some of the participants felt that the options and possibilities of the presented apps did not fit their personal needs in terms of their diabetes treatment. They expressed that important functions were missing (11/29, 38%), for example, the possibility to manage polypharmacy or comorbidity. Some also had the impression that existing features were irrelevant (10/29, 34%). A medication-related issue is illustrated in the following interview excerpt:

Medicament. The thing is, the older you get the more of this and this is added...

Participant 25

Yes.

Interviewer

If so, then it would somehow have to be here.

Participant 25

The different drugs.

Interviewer

Yes.

Participant 25

(The) possibility to enter an annotation, so that one can enter insulin dosages. Especially when being physically active, to be able to leave out a dosage, the value, because of this, that is naturally a bit interactively presented...

Participant 3

Nearly half of all participants (14/29, 48%) repeatedly stated that neither of the two tested apps offered an additional benefit to their regular diabetes treatment. Of the participants, 34% (10/29) stated that it would be too difficult and time-consuming to obtain the skills required to work with this technology.

### Positive Impressions During the Diabetes App Test

During the course of testing, the participants also reported positive impressions. Of 10 participants, 5 (50%) were of the opinion that using diabetes apps would have a positive effect on their self-reflection and the monitoring of their therapy. Of 10 participants, 2 (20%) were impressed by the clear arrangement and presentation of the entered data, as well as the simplicity and speed of the documentation. Both patient groups, those with and those without prior experience in using smartphones and tablets, reported positive impressions.

### Features and Design of a Useful Diabetes App

**Figure 3:**
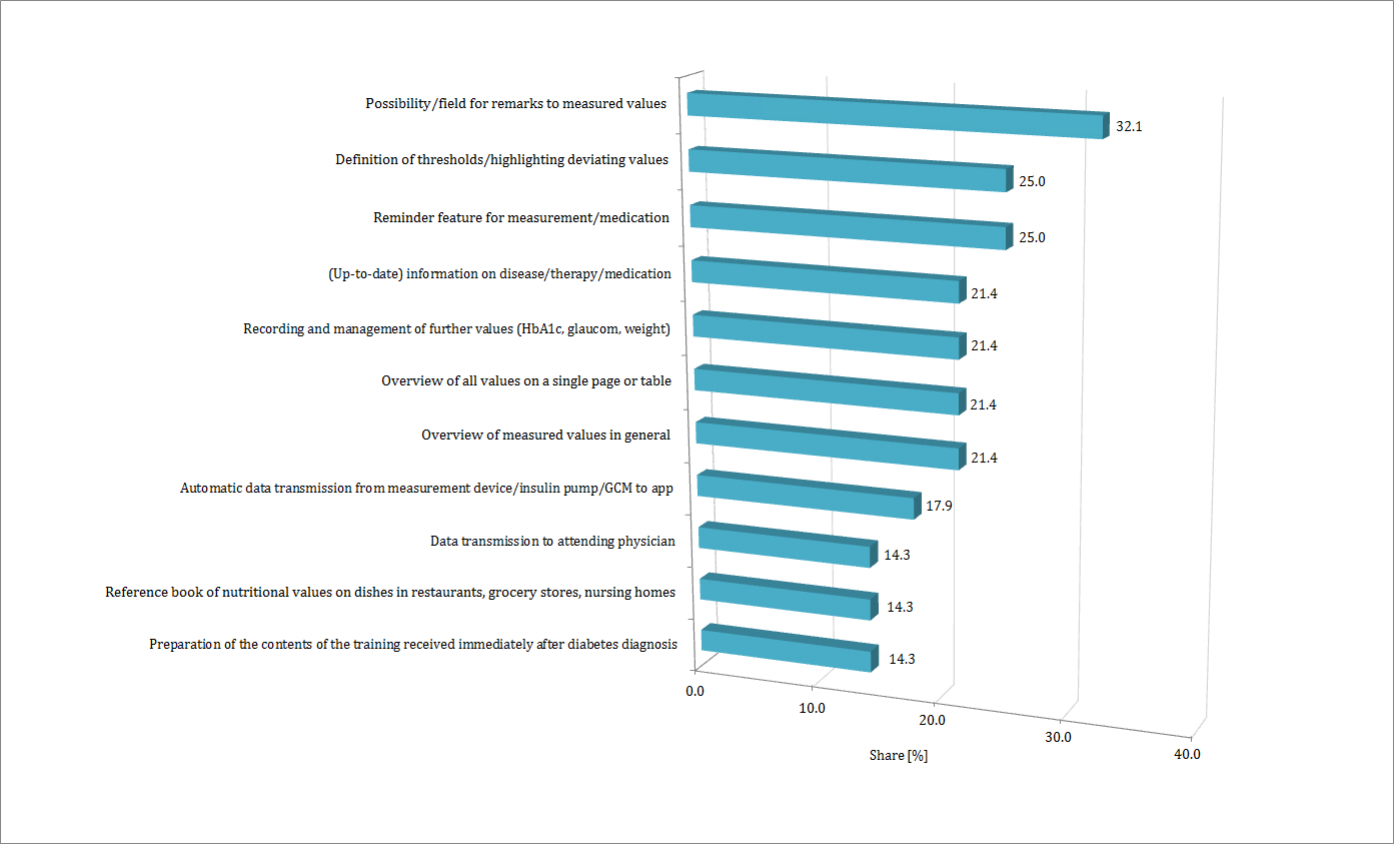
Ideas and needs for new diabetes apps (multiple selections possible, n=28).

In the course of this survey, we asked the participants what features they would expect to find in a helpful diabetes app (ie, which features would entail individual additional value). Of 28 participants, 9 (32%) expressed their need to be able to add personal remarks to the measured blood glucose values (see Figure 3). They said this would be useful should it be necessary to recreate the situation under which data was aggregated (ie, extremely low or high blood glucose values). A quarter of the participants (7/28, 25%) said they would appreciate a reminder feature for medication or blood glucose measurement. The same number of participants (7/28, 25%) felt that it would be useful to be able to define individual thresholds for blood glucose values and to be able to highlight deviating values. This is illustrated in the following quote:

It might be highlighted, if it is above or below, so that this here, if it is dangerous, is red and the rest could be yellow or something, so that I say, “Aha. That´s green, normal.” Just like a traffic light. Red, danger, and yellow is for or it decreases. Well. That would be good.

Participant 14

Of 28 participants, 6 (21%) would like to find a kind of reference book in which to look up information about diabetes mellitus, its treatment, and medication. Furthermore, they would appreciate finding information regarding nutritional facts for meals, which one would consume in a restaurant, or meals that have not been prepared by oneself (4/28, 14%). In order to support the documentation of values, participants would prefer to find all information (ie, blood glucose values, medication, and annotations) on a single page or table (6/28, 21%). This reveals their perception and preference of already existing and utilized blood glucose diaries. This is illustrated in the following quote:

You know, not that I have to click here again and another table (appears), and something else. And here, I know, with this small piece (authors note: patient presents blood glucose diary), I have the overview. If such a table would be in here I wouldn´t object to it.

Participant 6

Altogether, the tested apps did not provide any of the features the patients would expect to find in a helpful diabetes app, except for the reminder feature.

In addition to distinctive features, characteristics of the design of an app are crucial for their acceptance and usage among diabetics 50 years and older. The special usability requirements provided by elderly users of mobile apps have been presented and evaluated by Arnhold et al [1].

### Contact Persons for Technical Questions

For questions related to using modern technology—in general, or specifically for diabetes treatment—family members were the primary choice for 61% (19/31) of the participants, particularly their children, grandchildren, and partners. The second most frequently asked group of people were friends or people from their peer group (11/31, 35%). The least favorite source for information was the Internet or online forums used to provide solutions for technical difficulties (2/31, 6%).

## Discussion

### Principal Findings

#### Previous Knowledge and Experiences: Secondary Impact Factor

Regarding the treatment of diabetes patients aged 50 or older, apps only played a minor role among the participants. Only 2 out of 32 (6%) of the interviewees had already used diabetes apps. The Diabetes App Market Report [5] published in 2014 also illustrates that the affected patients accept and utilize diabetes apps rather restrainedly. Although the overall percentage (8/32, 25%) of smartphone and tablet users was significantly higher, it was still lower than that of neighboring countries such as Switzerland, where 52.0% of 55- to 69-year-olds are using this technology [52]. Even without consideration of age, Germany is far behind in the use of smartphones compared to Spain, Italy, Canada, the US, or the United Kingdom [53,54]. The survey showed that a lack of experience with handling smartphones and tablets minimizes the intention of using apps. However, increasing smartphone penetration will lead to a considerable increase in the experience of the target group (ie, those aged 50 or older) in handling such devices. Therefore, this obstacle for using those apps can be expected to diminish in the future.

#### Perceived Ease of Use: Main Impact Factor

The elderly have notably different requirements in terms of the handling of mobile apps compared to younger people. These have been presented, as well as evaluated, in the two previous substudies [1]. Taking those different usability requirements into consideration will immediately and positively impact the perceived ease of use [1]. As part of the survey, the app test showed that disregarding those usability requirements will result in the greatest impediment when (first) using the apps. The surveyed participants did not intuitively grasp the concept of how to execute the first steps (during first use). There were difficulties concerning the understanding of the menu guidance and navigation, the menu labelling, and the recognition of touch-sensitive areas of the screen. Additionally, barriers to ease of utilization were fonts and representations that were too small, as well as color contrast that was too low or absent. The obvious shortcomings in user friendliness of recently available apps for patients aged 50 or older have also been shown in studies by Arnhold et al [1], Schmid et al [16], and Grindrod et al [55]. However, even if apps reach a high degree of usability, it does not necessarily mean that elderly users will use the app intensively on a long-term basis within the scope of their therapy.

#### Perceived Additional Benefit: Main Impact Factor

Another main impact factor the study revealed was the perceived additional benefit for diabetes patients aged 50 or older. Therefore, it confirmed results of former studies regarding the acceptance of technology [16,18,39,55-57]. In conjunction with the perceived ease of use, these are the dominating impact factors on the acceptance of technology, both in the former studies and in this study. The lack of additional benefits is a considerable impact factor, which was revealed during the use of smartphones and tablets, as well as apps. This indicates that diabetes patients aged 50 or older are not sufficiently aware of the advantages provided by the apps when compared to previous types and methods of therapy management. Grindrod et al [55] have shown the same results within their usability study evaluating the perceptions of older adults concerning mobile medication management apps. Therefore, a diabetes app must provide clear benefits in comparison to conventional blood glucose diaries in written form (eg, for documenting purposes). Both types of documentation serve a practical purpose when being on the way, but several helpful functions were suggested that only a diabetes app could deliver: a reminder feature for medication/measurement, the definition of thresholds and the highlighting of deviating values, (current) information on disease/therapy/medication, and an automatic and wireless transmission of blood glucose data from the measurement device to the app or to the attending physician. These functions are similar to those that were found to be helpful in studies by Lorenz and Oppermann [9], Mallenius et al [8], and Schmid et al [16].

The target group of diabetes patients aged 50 or older is a rather heterogeneous one. For that reason, it is impossible to address the needs of all diabetes patients adequately with one diabetes app in order to gain an additional benefit. This was also shown during the survey. Specific functions of the tested apps were found to be irrelevant, or individually important ones were missing. Because of this, it is vital to take a modular and individually adjustable approach when developing and programming an app. Numerous studies also verified this to be a crucial aspect to app development [7,9,16,55]. Another possibility would be the implementation of autodidactic modules in an app, following and adapting to the learning process of the user.

#### Current State of Health: Secondary Impact Factor

The perceived additional benefit is closely linked to both the current state of health and the need for support (see section on perceived additional benefit). Accordingly, there are groups of patients that gain considerably more additional benefit by app usage compared to others. For instance, the effort of measurement, medication, and documentation presents a much bigger obstacle for diabetics with insulin therapy than for those who are treated with oral antidiabetics. It could be very useful to simplify and optimize these tasks with an electronic data transfer, graphical illustrations, or trend analysis. An additional option is the use of apps that support recently diagnosed type 2 diabetics, apps that can give information regarding the disease and therapy, and provide aid on how to change habits. In the best case scenario, this strategy could delay the beginning of a medical treatment. For this area of application, it would be sensible to have apps that could autonomously adapt to the needs of their users. This has been shown by Schmid et al [16], in which health apps that help manage daily therapy tasks and support health were found to be useful. Therefore, the results of this survey show the possible influence of health status on the additional benefit and, consequently, the acceptance of the technology.

Until now, with very few exceptions [58], there is a lack of well-directed integration of apps into individual treatment planning. The reason for that is the nonexistence of binding regulations in terms of documentation requirements, liability, and invoicing amongst attending physicians. Additionally, questions remain concerning insufficient interoperability and the integration of gathered data into health care systems [59,60].

#### Available Support: Secondary Impact Factor

When finding answers to questions regarding technology, a personal contact person plays a central role for diabetes patients aged 50 or older. The first choices for help were family members, especially children and grandchildren, followed by acquaintances and friends, the local distributor, and the attending physician. The importance of the family and acquaintances was demonstrated in a study by Mallenius et al [8]. The collection of information via the Internet, operating instructions, or service hotlines hardly played a role. Thus, if one tries to increase acceptance in the target group of diabetes patients aged 50 or older, there should be a personal introduction and a contact person in attendance during the initial phase of use (eg, the Amazon Mayday service). The results of this survey are supported by similar results from the MOBILE.OLD project [61] and by Schmid et al [16].

#### Trust in Own Technical Abilities/Insecurities in Utilization, Perceived Data Security, and Expected Reliability/Fault Tolerance: Secondary Impact Factors

Personal contact persons can have an advisory and clarifying role in terms of helping with diffuse insecurities in technology utilization. This survey showed that diabetes patients aged 50 or older fear data loss, insufficient data protection, and, especially, erroneous data input and its consequences. Taking into account that diabetes apps are dealing with health-related data, their fear is justified. The study by Schmid et al showed that the reliability of mobile apps is an essential acceptance factor. Thus, concerns regarding data security have a negative impact on acceptance [16]. The guarantee of data protection in cases of apps is a current key issue at the European and international level. Extensive attempts aim at defining, harmonizing, and implementing binding quality standards and regulations of certification [60].

#### Joy of Use: Secondary Impact Factor

Generally, the results of the survey showed that people with diabetes aged 50 or older are very open-minded regarding technology, thereby confirming the results of the investigations by Renaud and Biljon [19] and Steele et al [10]. Simultaneously, the reasons against the utilization of smartphones, tablets, and apps were collectively described as an “absence of joy of use.” This agrees with the results from studies by Kwon and Chidambaram [17], Conci et al [14], and Schmid et al [16]. For instance, the implementation of playful elements (ie, gamification) can increase pleasure and motivation during the utilization of the app. However, until now only a few diabetes apps make use of these elements, as shown by the Diabetes App Market Report [5].

### Limitations

When interviewing study participants, there is always the possibility that their answers are influenced by social desirability, which in turn could lead to biased results. To tackle this issue, we opted for an open interview setting, gave participants the chance to ask questions, and kept the number of people present during the interview to a minimum.

The choice of guided interviews as the method for our data collection was done with regard to an open interview setting and the chance to ask further targeted questions, in case particularly interesting or relevant topics arose. However, we restricted the evaluation of the gathered data to the unambiguous statements provided by participants during the interview. Naturally, we could not have drawn any conclusions on aspects and needs that might be relevant to the participants, but were not presented to us.

### Strengths

At the time of preparing this article, there were no other studies investigating the acceptance of diabetes apps by patients aged 50 or older. Hence, this study makes an essential contribution toward a better understanding of the promoting and inhibiting factors that influence the acceptance and usage of diabetes apps. We consciously decided to use a qualitative research method in order to have an open approach toward this field of research, and to put focus on the relevant subjective aspects of the participants. In combination with the work by Arnhold et al [1], it is now possible to conceptualize diabetes apps that are tailored to the needs, skills, and usability requirements of the target group, diabetics aged 50 or older. In addition, the results can be used as a starting point for quantitative studies in this field with a larger sample size.

### Conclusions

This study was the first to examine the factors that have an impact on the acceptance of mobile diabetes apps by patients aged 50 or older. The key factors that emerged for acceptance were the perceived additional benefit and the perceived ease of use. Less influential factors were previous experiences/knowledge, current health status, available support, trust in own technical abilities, perceived data security, expected reliability/fault tolerance, and joy of use. Furthermore, we showed that the needs of the investigated target group are highly heterogeneous due to differences in previous knowledge, age, type of diabetes, and therapy. Therefore, the contents of a helpful diabetes app should be individually adaptable. Personal contact persons, especially during the initial phase of use, are of utmost importance to reduce the fear of data loss or erroneous data input, and to raise acceptance among this target group.

## Multimedia Appendix 1

Overview of consulted studies related to the acceptance factors for diabetes apps.

## Multimedia Appendix 2

Interview guideline.

## Multimedia Appendix 3

Overview of interview results.

## Abbreviations

DIN: Deutsches Institut für Normung e.V.
ISO: International Organization for Standardization
MOPTAM: Mobile Phone Technology Acceptance Model
QDA: qualitative data analysis
STAM: Senior Technology Acceptance and Adoption Model
TAM: Technology Acceptance Model
TU: Technische Universität
UTAUT: Unified Theory of Acceptance and Use of Technology

## References

[1] Arnhold M, Quade M, Kirch W (2014). Mobile applications for diabetics: a systematic review and expert-based usability evaluation considering the special requirements of diabetes patients age 50 years or older. J Med Internet Res.

[2] International Diabetes Federation (2013). IDF Diabetes Atlas. 6th edition.

[3] (2014). Deutsche Diabetes-Forschungsgesellschaft eV Düsseldorf.

[4] Heidemann C, Du Y, Scheidt-Nave C, Robert Koch-Institut (2011). Diabetes mellitus in Deutschland. GBE kompakt.

[5] Jahns R-G (2014). research2guidance.

[6] Holzinger A, Searle G, Nischelwitzer A (2007). On some aspects of improving mobile applications for the elderly. UAHCI'07 Proceedings of the 4th International Conference on Universal Access in Human Computer Interaction: Coping with Diversity.

[7] Lorenz A, Oppermann R, Zahl L, Mielke D (2007). Personalized mobile health monitoring for elderly. MobileHCI ’07 Proceedings of the 9th International Conference on Human Computer Interaction with Mobile Devices and Services.

[8] Mallenius S, Rossi M, Tuunainen VK (2007). http://classic.marshall.usc.edu/assets/025/7535.pdf.

[9] Lorenz A, Oppermann R (2009). Mobile health monitoring for the elderly: designing for diversity. Pervasive Mob Comput.

[10] Steele R, Lo A, Secombe C, Wong YK (2009). Elderly persons' perception and acceptance of using wireless sensor networks to assist healthcare. Int J Med Inform.

[11] Davis FD, Bagozzi RP, Warshaw PR (1989). User acceptance of computer technology: a comparison of two theoretical modells. Manage Sci.

[12] Malhotra Y, Galletta DF (1999). Extending the technology acceptance model to account for social influence: theoretical bases and empirical validation. HICSS-32. Proceedings of the 32nd Annual Hawaii International Conference on Systems Sciences.

[13] Ziefle M, Bay S (2005). How older adults meet complexity: aging effects on the usability of different mobile phones. Behav Inf Technol.

[14] Conci M, Pianesi F, Zancanaro M, Gross T, Gulliksen J, Kotzé P, Oestreicher L, Palanque P, Prates RO, Winckler M (2009). Useful, social and enjoyable: mobile phone adoption by older people. Human-Computer Interaction – INTERACT 2009.

[15] Kleinashi M, Hiyama A, Miura T, Campos P, Graham N, Jorge J, Nunes N, Palanque P, Winckler M (2011). Elderly user evaluation of mobile touchscreen interactions. Human-Computer Interaction – INTERACT 2011.

[16] Schmid A, Dörfler I, Dany F, Böpple O, Shire KA, Leimeister JM (2012). Analyse der Akzeptanzkriterien für mobile Anwendungen im Bereich Gesundheit in der Zielgruppe 50+. Technologiegestützte Dienstleistungsinnovation der Gesundheitswirtschaft.

[17] Kwon HS, Chidambaram L (2000). A test of the technology acceptance model: the case of cellular telephone adoption. Proceedings of the 33rd Hawaii International Conference on System Sciences.

[18] Venkatesh V, Morris MG, Davis GB, Davis FD (2003). User acceptance of information technology: toward a unified view. MIS Quarterly.

[19] Renaud K, Van Biljon J (2008). Predicting technology acceptanceadoption by the elderly: a qualitative study. SAICSIT '08 Proceedings of the 2008 Annual Research Conference of the South African Institute of Computer Scientists and Information Technologists on IT Research in Developing Countries: Riding the Wave of Technology.

[20] Legris P, Ingham J, Collerette P (2003). Why do people use information technology? A critical review of the technology acceptance model. Information & Management.

[21] Ellis D, Allaire JC (1999). Modeling computer interest in older adults: the role of age, education, computer knowledge, and computer anxiety. Hum Factors.

[22] Brandt M, Voß B, Voß R (2002). Abschlussbericht zur Studie: Analyse der Determinanten der Technikaufgeschlossenheit und des Nachfrageverhaltens in Bezug auf seniorengerechte Technik - untersucht in den Anwendungsbereichen Mobilität, Sicherheit, Kommunikation, Wohnungsgestaltung und Haushalt.

[23] Van Biljon J, Kotzé P (2007). Modelling the factors that influence mobile phone adoption. SAICSIT '07 Proceedings of the 2007 Annual Research Conference of the South African Institute of Computer Scientists and Information Technologists on IT Research in Developing Countries.

[24] Arning K, Ziefle M (2007). Understanding age differences in PDA acceptance and performance. Comput Human Behav.

[25] Lee YS (2007). Older Adults' User Experience With Mobile Phones: Identification of User Clusters and User Requirements [dissertation].

[26] Lu J, Yu C, Liu C, Yao JE (2003). Technology acceptance model for wireless Internet. Internet Research.

[27] Kaasinen E (2005). User Acceptance of Mobile Services - Value, Ease of Use, Trust and Ease of Adoption [thesis].

[28] Nysveen H, Thorbjernsen H (2005). Intentions to use mobile services: antecedents and cross-service comparisons. J Acad Market Sci.

[29] Holzinger A, Searle G, Kleinerberger T, Seffah A, Javahery H, Miesenberger K, Klaus J, Zagler W, Karshmer A (2008). Investigating usability metrics for the design and development of applications for the elderly. Computers Helping People with Special Needs. Lecture Notes in Computer Science.

[30] Alagöz F, Wilkowska W, Roefe D, Klack L, Ziefle M, Schmitz-Rode T (2010). Technik ohne Herz? Nutzungsmotive und Akzeptanzbarrieren medizintechnischer Systeme aus der Sicht von Kunstherzpatienten.

[31] Gaul S, Ziefle M, Wilkowska W, Arning K, Kasugai K, Röcker C, Jakos EM (2010). Technikakzeptanz als integraler Bestandteil der Entwicklung medizintechnischer Produkte.

[32] Holden RJ, Karsh BT (2010). The technology acceptance model: its past and its future in health care. J Biomed Inform.

[33] Schaar AK, Ziefle M, Groß D, Gründer G, Simonovic V (2010). Technikakzeptanz und nutzenbewertungen im kontext neuartiger medizintechnischer anwendungen. Akzeptanz, Nutzungsbarrieren und Ethische Implikationen Neuer Medizintechnologien. Die Anwendungsfelder Telemedizin und Inkorporierte Technik.

[34] Banse M (2008). Softwareergonomische Optimierung Touchscreen-Basierter Mensch-Computer-Interaktion.

[35] Leonardi C, Albertini A, Pianesi F, Zancanaro M (2010). An exploratory study of a touch-based gestural interface for elderly. NordiCHI '10 Proceedings of the 6th Nordic Conference on Human-Computer Interaction: Extending Boundaries.

[36] Caprani N, O’Connor NE, Gurrin C (2012). Touch screens for the older user.

[37] Kleinberger T, Becker M, Ras E, Holzinger A, Müller P, Stephanidis C (2007). Ambient intelligence in assisted living: enable elderly people to handle future interfaces. Universal Access in Human Computer Interaction. Ambient Interaction. Lecture Notes in Computer Science.

[38] Niemelä M, Fuentetaja RG, Kaasinen E, Gallardo JL, Schiele B, Dey AK, Gellersen H, de Ruyter B, Tscheligi M, Wichert R, Aarts E, Buchman A (2007). Supporting independent living of the elderly with mobile-centric ambient intelligence: user evaluation of three scenarios. Ambient Intelligence. Lecture Notes in Computer Science.

[39] Peek ST, Wouters EJ, van Hoof J, Luijkx KG, Boeije HR, Vrijhoef HJ (2014). Factors influencing acceptance of technology for aging in place: a systematic review. Int J Med Inform.

[40] Deutscher Bundestag (2010). BT-Drucksache 17/3815 vom 17.11.2010: Sechster Bericht zur Lage der älteren Generation in der Bundesrepublik Deutschland - Altersbilder in der Gesellschaft.

[41] Deutsches Institut für Normung (2003). DIN-Fachbericht 131: Leitlinien für Normungsgremien zur Berücksichtigung der Bedürfnisse von älteren Menschen und von Menschen mit Behinderungen.

[42] International Organization for Standardization (IOS), International Electrotechnical Commission (IEC) (2000). http://www.iso.org/iso/iso_iec_gen3_2000-en.pdf.

[43] International Organization for Standardization (IOS), International Electrotechnical Commission (IEC) (2001). http://www.iso.org/iso/iso_iec_guide_71_2001.pdf.

[44] Holzinger A, Miesenberger K, Klaus J, Zagler W (2002). User-centered interface design for disabled and elderly people: first experiences with designing a Patient Communication System (PACOSY). Computers Helping People with Special Needs. Lecture Notes in Computer Science.

[45] Phang CW, Sutanto J, Kankanhalli A, Li Y, Tan BCY, Teo HH (2006). Senior citizens' acceptance of information systems: a study in the context of e-government services. IEEE Trans Eng Manage.

[46] Holzinger A, Schaupp K, Eder-Halbedl W, Miesenberger K, Klaus J, Zagler W, Karshmer A (2008). An investigation on acceptance of ubiquitous devices for the elderly in a geriatric hospital environment: using the example of person tracking. Computers Helping People with Special Needs. Lecture Notes in Computer Science.

[47] Tolar M (2008). Assistive Technologien. Studie im Auftrag des Bundeskanzleramts. Endbericht.

[48] Lux P, Mueller T, Burkhard M (2012). Android Tablet-Computer im Pilottest mit Senioren.

[49] Mayring P (2010). Qualitative Inhaltsanalyse. Grundlagen und Techniken. 11th edition.

[50] Kuckartz U (2010). Einführung in die Computergestützte Analyse qualitativer Daten. 3rd edition.

[51] Mayer HO (2009). Interview und Schriftliche Befragung: Entwicklung, Durchführung und Auswertung.

[52] YR (2013). Besitz von Mobilen Endgeräten in der Schweiz nach Altersgruppen im Jahr 2013.

[53] (2013). ComScore.

[54] (2013). ComScore.

[55] Grindrod KA, Li M, Gates A (2014). Evaluating user perceptions of mobile medication management applications with older adults: a usability study. JMIR Mhealth Uhealth.

[56] Davis FD, Bagozzi RP, Warshaw PR (1989). User acceptance of computer technology: a comparison of two theoretical models. Manage Sci.

[57] Van Biljon J, Renaud K, Song IY, Piattini M, Chen YPP, Hartmann S, Grandi F, Trujillo J, Opdahl AL, Ferri F, Grifoni P, Caschera MC, Rolland C, Woo C, Salinesi C, Zimányi E, Claramunt C, Frasincar F, Houben GJ, Thiran P (2008). A qualitative study of the applicability of technology acceptance models to senior mobile phone users. Advances in Conceptual Modeling – Challenges and Opportunities.

[58] Central Krankenversicherung AG (2014). Die initiative.diabetes.

[59] World Health Organization (WHO) (2009). Telemedicine: Opportunities and Developments in Member States. Report on the Second Global Survey on eHealth.

[60] Europäische Kommission (2014). Grünbuch über Mobile-Health-Dienste (“mHealth”).

[61] Spiru L, Karlhuber I, Turcu I, Van der Vaart N, Schurz S, Laperal J (2014). Advanced Technology Services for Supporting Active Seniors: the Mobile.Old Project.

