# Supplementary material for: Acceptance Factors of Mobile Apps for Diabetes by Patients Aged 50 or Older: A Qualitative Study
Source: Med 2 0. 2015 Mar 2;4(1):e1. doi: 10.2196/med20.3912 (PMC4376102; doi:10.2196/med20.3912)
Supplement: Supplementary file 1 [file med20_v4i1e1_app1.pdf]

**Topic**	**Studies**
Predictors for the acceptance of technology in general	[11, 12, 20]
Predictors for the acceptance of technology in general for people aged 50 or older	[19, 21, 22]
Predictors for the acceptance of mobile devices (cellular phone, PDA, tablet) in general	[17, 23]
Predictors for the acceptance of mobile devices (cellular phone, PDA, tablet) for people aged 50 or older	[8, 13, 14, 19, 24, 25]
Predictors for the acceptance of mobile services and the Internet in general	[26, 27, 28]
Predictors for the acceptance of mobile services and the Internet in general for people aged 50 or older	[6, 8, 9, 9, 29]
Predictors for the acceptance of mobile services and/or apps within the field of health care	[10, 16]
Predictors for the acceptance of medical technology in general	[30, 31, 32, 33]
Predictors for the acceptance of touchscreen based technology for people aged 50 or older	[15, 34, 35, 36]
Concept of Ambient Assisted Living<br>(= age-appropriate assistance systems) and their acceptance for people aged 50 or older	[37, 38, 39]
Studies/reports concerned with the changes taking place during the aging process, ie the living and health situation of the elderly	[40, 41, 42, 43]
Additional topic related studies	[44, 45, 46, 47]
