# Supplementary material for: Acceptance Factors of Mobile Apps for Diabetes by Patients Aged 50 or Older: A Qualitative Study
Source: Med 2 0. 2015 Mar 2;4(1):e1. doi: 10.2196/med20.3912 (PMC4376102; doi:10.2196/med20.3912)
Supplement: Supplementary file 2 [file med20_v4i1e1_app2.pdf]

Interview guideline

Socio-demography

- Sex m/f? (Do not ask: Enter autonomously!)
- May I begin by asking about your age?
- What is the highest educational qualification you have completed?
- What is your current occupation, or the last occupation you worked in?

Medical history/current therapy

- When was your diabetes mellitus diagnosed?
- Which type of diabetes mellitus was diagnosed for you?
- How is your diabetes mellitus currently being treated?

Open-mindedness towards technical devices in the diabetes therapy

- What´s your general stance on available new technical devices designed for the therapy of diabetes?
    - If: I am not interested
        - Why doesn´t it interest you?

Current implementation of technical devices in the diabetes therapy

- Which devices do you currently use to help with your diabetes mellitus therapy?

Utilisation of mobile devices and apps in general, and for diabetes mellitus treatment

Reasons for or against the utilisation of mobile devices/apps

- Do you own a smartphone or tablet?
    - NO – Why not?
    - YES – Would you describe using such a device as easily comprehendible?
        - Do you know what the term „App“ stands for?
            - NO
            - YES – Do you use apps?
                - NO – Why haven´t you used apps yet?
                - YES

- Do you use apps specifically for the purpose of supporting your diabetes treatment?
  - NO – Why haven´t you tried using apps that support the diabetes treatment yet?
  - YES – Which app do you use?
    - Why did you decide to use this app specifically?

Required help when using current technology

- Should you require help concerning your mobile phone, smartphone or tablet, who do you turn to or where do you try to find information?

Content of a useful diabetes app

- Which features should an app provide in order to be of use in your daily treatment?

Test of two existing diabetes apps

- What is your first impression of the app?
- How would you assess the design of the colours, the colour contrast, the text size and the basic design of the app?
- Please try to enter data into the blood-glucose diary.
- Do you think that the display of the gathered data is helpful? What do you specifically like or dislike?
- How would you assess the graphical representation of the measured data? What do you specifically like or dislike?
- Do you feel that important features are missing? If so, which ones?
- Would you say that some features of this app are unnecessary? If so, which ones?
- Do you feel comfortable navigating through this app? Which aspects were easy, which were complicated?
- Do you have any further impressions concerning the app that you would like to share?

Further annotations

From your point of view, are there any relevant aspects or questions that you feel should be addressed, but weren´t mention thus far? Is there anything you would like to add?
